# Antimicrobial Activity of Olive Leaf Extract to Oral *Candida* Isolates

**DOI:** 10.3390/microorganisms12081726

**Published:** 2024-08-21

**Authors:** Maja Kinkela Devčić, Igor Pasković, Zoran Kovač, Petra Tariba Knežević, Luka Morelato, Irena Glažar, Sunčana Simonić-Kocijan

**Affiliations:** 1 Department of Oral Surgery, Faculty of Dental Medicine, University of Rijeka, Kresimirova 40/42, 51000 Rijeka, Croatia; maja.kinkela.devcic@fdmri.uniri.hr (M.K.D.); morelatoluka@gmail.com (L.M.); 2 Department of Agriculture and Nutrition, Institute of Agriculture and Tourism, K. Huguesa 8, 52440 Porec, Croatia; paskovic@iptpo.hr; 3 Department of Prosthodontics, Faculty of Dental Medicine, University of Rijeka, Kresimirova 40/42, 51000 Rijeka, Croatia; zoran.kovac@icloud.com (Z.K.); petra.tariba@fdmri.uniri.hr (P.T.K.); 4 Department of Oral Medicine, Faculty of Dental Medicine, University of Rijeka, Kresimirova 40/42, 51000 Rijeka, Croatia

**Keywords:** antifungal activity, *Candida* spp, olive leaf extract

## Abstract

Objectives: The aim of this study was to determine the antifungal activity of olive leaf extract (OLE) and the synergistic effect of standard antifungal therapy and OLE against clinical oral *Candida* species’ isolates. Materials and Method: The susceptibility of 60 clinical isolates of the *Candida* species (36 *C. albicans*, 16 *C. krusei*, 5 *C. glabrata* and 3 *C. tropicalis*) was tested with four concentrations of OLE (60 µg/µL, 120 µg/µL, 240 µg/µL and 333 µg/µL) and the synergistic effect of standard antifungal therapy and OLE (miconazole (MIC) + 333 µg/µL OLE and nystatin (NYS) + 333 µg/µL OLE). The antimicrobial activity was tested using the disk diffusion method. Results: All concentrations (60 µg/µL, 120 µg/µL, 240 µg/µL and 333 µg/µL) of OLE showed a statistically significant effect on all *Candida* species compared to the control (DMSO) except for the lowest concentration (60 µg/µL) tested on *C. glabrata*. There was a dose-dependent effect of OLE on tested samples. Concentrations of 240 µg/µL and 333 µg/µL showed statistically significant higher antifungal activity compared to the lowest concentration of 60 µg/µL. No statistically significant synergistic effect of OLE and standard antifungal therapy was found compared with standard therapy alone. Conclusions: The results of this study present the significant antimicrobial effect of OLE against all tested *Candida* species except for the lowest concentration on *C. glabrata*. Increasing the concentration of OLE also increases its effect on *Candida* species. This indicates the possible potential effect of OLE in the treatment of *Candida*-related oral diseases.

## 1. Introduction

*Candida* species (*Candida* spp.) are a part of the normal flora in the oral cavity [1]. They may cause opportunistic infections influenced by a number of factors that alter the oral environment and affect the resistance of the oral mucosa such as host immune status, antibiotic use, smoking, age, pregnancy, oral hygiene and the wearing of mobile prosthetic replacements [2]. *Candida albicans* appears to be the most common species. Other species that can be isolated from the oral cavity are *Candida tropicalis*, *Candida glabrata*, *Candida parapsilosis*, *Candida krusei* and *Candida pseudotropicalis* [3]. *Candida* may cause various forms of infections, from superficial to systemic conditions. Despite the development of new antifungal drugs and research into alternative approaches in the treatment of oral fungal infections [4,5,6], miconazole and nystatin remain the drugs of choice in the treatment of oral candidiasis primarily because of their clearly defined usefulness and low prices [7]. With the increasing use of antifungal agents, a growing number of resistant strains are emerging, which causes difficulties in the treatment of fungal infections of the oral cavity [8]. Therefore, many researchers are working to find new potential herbal therapeutics that may be a safer alternative in treatment [9]. Among natural products, olive leaf extract is a promising therapeutic tool for the treatment of oral infections. Many potentially bioactive substances in olive leaves can have antioxidant, antimicrobial, antihypertensive, antiviral, anti-inflammatory, hypoglycemic, neuroprotective and anticancer properties [10]. These properties are significantly related to the high amount of phenolic and secoiridoid composition of OLE extracts [11,12], which can vary depending on factors such as age or degree of leaf maturity, geographical origin, moisture content and degree of soil pollution [13]. Oleuropein and hydroxytyrosol are major constituents of olive leaf extracts and have an effect against a large number of pathogenic microorganisms [14,15]. Oleuropein increases the production of nitric oxide in macrophages and thereby contributes to the activity against infectious agents. It has been proven that oleuropein, along with other phenolic compounds, inhibits the growth of *Candida albicans* and *Candida dubliniensis* [16]. Therefore, the present study was designed to investigate the in vitro activity of OLE against oral *Candida* isolates and to test whether combined administration of OLE with standard therapy could be a more efficient alternative to standard therapy alone.

## 2. Materials and Methods

### 2.1. Clinical Oral Sampling

Oral samples from 45 patients (*n* = 45) who attended the Clinical Hospital Center Rijeka were used in this study. Regarding gender, 11 (24.44%) were men and 34 (75.55%) were women. The median age of the respondents was 71 years (interquartile range from 63 to 80 years) with a range from 45 to 83 years. Exclusion criteria were the use of antibiotics, antimycotics and antiseptics in the last 30 days, smoking tobacco products and predisposing systemic factors for increased growth of *Candida* in the oral cavity such as immunodeficiency, organ transplantation, AIDS/HIV, diabetes, malignant diseases and autoimmune diseases. *Candida* isolates were collected by the concentrated rinse method. Briefly, patients were instructed to rinse the oral cavity with 10 mL of sterile saline, and samples of the rinse solution were collected. From the collected samples, a concentrated rinse solution was prepared by centrifugation at 3500/rpm for 20 min. After removing the supernatant, the cell pellet was resuspended in 500 μL saline, and 100 μL of the sample was inoculated onto Chromagar Candida medium (CHROMagar, Paris, France). Patients were informed of the aims and purpose of this investigation and signed the informed consent for participation. This investigation was previously approved by both the Ethics Committee of the Clinical Hospital Center Rijeka and the Faculty of Dental Medicine in Rijeka, Croatia.

### 2.2. Candida Identification

Chromagar Candida was used for the growth and identification of the *Candida* species. Immediately after collecting the microbiological samples, they were planted directly on the Chromagar Candida chromogenic medium that differentiated five types of *Candida* (*C. albicans*, *C. krusei*, *C. glabrata*, *C. tropicalis* and *Candida* other species). After incubation (37 °C for 48 h), colonies of size 2–3 mm with smooth and shiny surfaces and a characteristic yeast smell were obtained. Colonies were differentiated and counted using a visual technique. *C. albicans* were identified as light green colored smooth colonies, *C. tropicalis* appeared as blue to metallic blue colored colonies, *C. glabrata* appeared as mauve–brown smooth colonies, while *C. krusei* appeared as pink fuzzy colonies. Isolates that produced white to mauve colonies were considered for *Candida* other species. After identification, the Candida colonies were transferred and stored in sterile containers containing a solution of brain heart infusion broth (Sigma-Aldrich, St. Louis, MO, USA) and 50% glycerol and were frozen at a temperature of −20 °C until further analysis.

### 2.3. Antifungal Susceptibility Test

The disk diffusion method was used to evaluate the susceptibility of oral *Candida* species to OLE and standard therapy. For standard therapy, nystatin 100 IU (Oxoid, Basingstoke, UK) and miconazole 50 µg (Oxoid, UK) commercial discs were used. To test the OLE effect, crude commercial OLE (Magdis, Zagreb, Croatia) in dry powder form was used. Olive leaf extract was prepared by a water extraction procedure. OLE was dissolved in sterile DMSO (Sigma-Aldrich) in concentrations of 60 µg/µL, 120 µg/µL, 240 µg/µL and 333 µg/µL. Blank discs with 6 mm diameters (Oxoid, UK) were impregnated with 10 µL of OLE solution of each concentration. For testing the possible synergic effect of standard therapy and OLE, commercial nystatin (100 IU) and miconazole discs (50 µg) were impregnated with 10 µL of OLE (333 µg/µL). Blank discs impregnated with 10 µL of sterile DMSO were used as a control. The disk diffusion method was performed according to the Clinical and Laboratory Standards Institute (CLSI)’s guidelines [17]. The susceptibility test was performed on clinical isolates of *C. albicans*, *C. krusei*, *C. glabrata* and *C. tropicalis.* All tests were performed in duplicate. Previously collected and frozen samples of *Candida* species were regrown on Chromagar Candida. Fresh colonies and sterile saline were used to prepare the standardized inoculum. The inoculum concentration was adjusted using a turbidimeter to 0.5 on the McFarland scale. Mueller–Hinton agar (Himedia laboratories, Mumbai, India) with 2% glucose and 0.5 μg/mL methylene blue was used for antifungal testing. A total of 200 µL of inoculum was distributed over the entire surface of the agar with a pipette and a sterile cotton swab. After 15 min, discs were placed on the plates. The plates were marked according to the protocol and placed in a thermostat at a temperature of 37 °C for 24 h. After incubation, the inhibition zones were measured and expressed in millimeters.

### 2.4. Determination of Polyphenols by HPLC

As described in [18], an analysis of olive leaf phenols in OLE was conducted using high-performance liquid chromatography (HPLC) with simultaneous UV/Vis detection at four different wavelengths on the Thermo Ultimate 3000 HPLC system (Thermo Fisher Scientific in Waltman, MA, USA). The olive leaf extract dry powder was dissolved in a mixture of methanol and water (8:2) and ultrasonicated for 20 min. The resulting mixture underwent centrifugation and filtration using a cellulose acetate syringe filter with 0.45 μm pores. We also employed a detailed set of HPLC conditions, as specified in [18]. To identify and quantify phenolic compounds, we compared their retention times with those of pure standards and utilized the external standard method along with corresponding calibration curves. Standards of oleuropein, luteolin-7-*O*-glucoside, luteolin-4-*O*-glucoside, apigenin-7-*O*-glucoside, verbascoside and rutin (HPLC grade) were purchased from Extrasynthese (Genay, France) and a standard of oleacein was purchased from Merck (Darmstadt, Germany).

### 2.5. Statistical Analysis

The normality of the distribution of numerical variables was tested by the Shapiro–Wilk test. Numerical data were described by the median and the limits of the interquartile range. Differences between two independent groups were tested by Mann–Whitney’s U test (Hodges–Lehmann median difference and 95% CI of difference). The differences between three or more groups were tested by the Kruskal–Wallis test (post hoc Conover). All *p* values were two-sided. The significance level was set to Alpha = 0.05. The statistical analysis was performed using MedCalc^®^ Statistical Software version 20.218 (MedCalc Software Ltd., Ostend, Belgium; https://www.medcalc.org; accessed on 15 April 2023).

## 3. Results

### 3.1. Candida Isolates

From 45 clinical oral samples, we isolated and identified, by Chromagar Candida, 36 *C. albicans*, 16 *C. krusei*, 5 *C. glabrata* and 3 *C. tropicalis*. From the samples of 13 patients, we identified both *C. albicans* and *C. krusei* and from 3, we identified *C. albicans* and *C. glabrata*. From 28 samples, we identified only 1 species (20 *C. albicans*, 3 *C. krusei*, 2 *C. glabrata* and 3 *C. tropicalis*) and from 1 clinical sample, we did not isolate *Candida*.

### 3.2. Analysis of Biophenols

Determination of polyphenols by HPLC analysis in the OLE identified the following compounds: oleuropein, luteolin-7-*O*-glucoside, oleacein, verbascoside, apigenin-7-*O*-glucoside, rutin and luteolin-4-*O*-glucoside (Table 1). The analyses confirmed that oleuropein is the most abundant phenol in the OLE, following the latest research on the presence of individual phenolic components in the olive leaf of different varieties [19].

### 3.3. Susceptibility of Candida Isolates

Statistically significant differences in the zones of inhibition of all tested concentrations of OLE compared to the control (DMSO) were recorded except for the concentration of 60 µg/µL on *C. glabrata* (Table 2).

There were statistically significant differences in *C. albicans* and *C. krusei* due to the used concentrations of OLE. OLE concentrations of 240 µg/µL and 333 µg/µL had a significantly better effect on *C. albicans* (Kruskal–Wallis test, *p* = 0.006) and *C. krusei* (Kruskal–Wallis test, *p* = 0.02) compared to the concentration of 60 µg/µL (Table 3).

Comparing the synergic effect of standard therapy and OLE with standard therapy alone, there was no significant difference in the zone of inhibition. Neither the nystatin + OLE (333 µg/µL) or miconazole + OLE (333 µg/µL) showed differences compared with miconazole or nystatin alone. (Table 4 and Table 5).

## 4. Discussion

The occurrence of fungal resistance to antifungal drugs leads to difficulties in treating fungal infections of the oral cavity and prompted doctors to change their approach to treating fungal infections. The number of species showing intermediate or complete drug resistance is increasing, primarily due to the increased use of antifungal drugs. In addition to resistance, other factors limit the use of available antifungal drugs, such as the inadequate spectrum of action, poor bioavailability, low tolerance index, interactions with other drugs, inappropriate pharmacokinetic profile and significant toxic effects [20,21,22].

Many researchers are engaged in finding new potential herbal therapeutics that can represent a safer alternative to fungal infection treatment [23]. Combination therapy is another way to increase the effectiveness of antifungal drugs and reduce their potential toxic effects. By combining the pharmacological properties of conventional drugs with bioactive compounds present in plant extracts, synergism can lead to increased efficacy against microbial infections. This approach enhances antimicrobial activity and reduces the likelihood of developing drug resistance. Also, synergistic combinations offer the potential to reduce required drug doses, reducing the risk of toxicity and side effects in patients. Investigating synergistic interactions between drugs and plant extracts represents a promising avenue for optimizing antimicrobial therapies and improving patient outcomes.

We investigated the possibility of treating fungal infections from natural sources such as phenolic compounds from olive-based preparations. Previous research has established the antifungal activity of phenolic compounds through the inhibition of cell growth, that is, damage to the cell membrane of the fungus; however, the mechanism of the antimicrobial effect of phenol oleuropein has not been fully elucidated [24]. Some authors believe that the ortho-diphenol system (catechol) in the molecular structure of phenol oleuropein is responsible for the mentioned effect [25]. While oleuropein is a significant contributor to the antimicrobial activity of OLE, the presence of other phenolics amplifies this effect, making the crude extract more potent than isolated compounds. This synergism is thought to occur because the various phenolics can disrupt microbial cell membranes, inhibit enzyme activity and interfere with microbial metabolism in complementary ways [26]. The combination of phenolic compounds often results in a synergistic effect, enhancing the overall antimicrobial efficacy of the extract.

Analysis of *Candida* exposure to OLE showed a concentration-dependent effect. Intergroup comparison showed statistically significant differences between the tested concentrations. *C. albicans* and *C. krusei* treated with concentrations of 240 µg/µL and 333 µg/µL had a significant effect compared to the lowest concentration (60 µg/µL). The results are in accordance with the results of Zorić et al. [16], who demonstrated the concentration-dependent effects of OLE on *C. albicans* and *C. dubliniensis*. Also following the mentioned research, all our tested concentrations (60 µg/µL, 120 µg/µL, 240 µg/µL and 333 µg/µL) were significantly more effective compared to the control except for the lowest concentration of 60 µg/µL on *C. glabrata.* In other research by Zorić et al. [27], they demonstrated that oleuropein as a component of olive leaf causes a decrease in the total content of ergosterol in the cell membrane of *C. albicans*, which could be involved in the mechanism of antifungal action. Additionally, other researchers have investigated the impact of OLE on *Candida* species. For instance, Markin et al. [28] observed that OLE destroyed 15% of *C. albicans* within 24 h. Pereira et al. [15] also performed a study to analyze the antimicrobial activity of olive leaf extracts and screened the inhibitory effects against some Gram-positive and Gram-negative bacteria and *C. albicans* where the most sensitive microorganisms were *B. cereus* and *C. albicans*.

We tested the effectiveness of OLE in combination with miconazole and nystatin. The concentrations of OLE that were used to test the synergistic effect were based on those used in clinical therapy [29]. Olive leaf extract in combination with miconazole and nystatin had an indifferent effect compared to the standard drug used alone. It may be the result of no existing standard procedures or methods for determining the sensitivity of yeasts to plant extracts [30] and determining synergism with antifungal drugs. Antimicrobial test systems should be simple, fast, reproducible and inexpensive, and the disc diffusion method has often been used precisely for these reasons. Its accessibility and ease of implementation have facilitated numerous studies, enabling researchers to swiftly evaluate the antimicrobial activity of various substances, including plant extracts. Abd-Elmonsef et al. [31], in their research, used the disc diffusion method to prove a significant synergistic inhibitory effect of plant extracts, the main component of which is the polyphenolic system, combined with antifungal drugs on *Candida*. Avijgan et al. [32], also using the disk diffusion method, proved the synergistic effect of plant extracts and azoles on clinical isolates of *Candida*. Also, the precision and reliability of the disc diffusion method have been demonstrated through numerous studies, showcasing its effectiveness in assessing antimicrobial susceptibility when compared to alternative methods [33,34,35]. A.L. Barry at. al. [36] concluded in their research that susceptibility testing by the disc diffusion method was just as reproducible as the results of the microdilution procedure. Furthermore, the precision of disc diffusion assays was maximized by using Muller–Hinton agar with glucose and methylene blue and by incubating the discs for only 24 h. E. Lopez-Oviedo et al. [37] demonstrated the disc diffusion method as an attractive method for determining susceptibility because of its simplicity, low cost and ability to obtain the results earlier because it is possible to record the reading after 24 h of incubation. However, the absence of an inhibition zone when testing plant extracts does not necessarily mean that the compound is inactive, especially for compounds that diffuse more slowly into the culture medium [38] and those that are difficult to dissolve or insoluble in water and thus their hydrophobic nature prevents uniform diffusion through agar media [39].

All isolates collected in this research showed susceptibility to standard therapy with miconazole and nystatin, which is in accordance with the research of Carvalhinho et al. [40], who noted the sensitivity of all isolates to miconazole, and 35% of the isolated strains were sensitive to miconazole depending on the applied dose. The number of strains showing resistance to antifungal drugs is increasing. Kuriyama et al. [41] determined the sensitivity of most isolates to miconazole, although 14.3% of *C. krusei* isolates showed resistance. Similar results were obtained by Manfredi et al. [42], where as many as 21% of the isolates showed intermediate resistance. Although, the mentioned researches show a growing trend of development of resistance, in our study, none of the isolates showed resistance to standard therapy. This may be the reason for the absence of a significant synergistic inhibitory effect of OLE and standard therapy. Testing synergism on resistant strains is the most important to determine the effectiveness of olive leaf compounds fully. By screening these resistant strains, we can elucidate whether olive-based preparations have the potential to overcome drug resistance mechanisms, thereby expanding their therapeutic utility. Such research supports the inclusion of olive leaf compounds in antimicrobial therapies, particularly in the fight against emerging drug-resistant pathogens. Furthermore, the evaluation of synergistic effects on resistant strains provides valuable insight into potential mechanisms of action, offering a deeper understanding of how olive-derived compounds interact with microbes to exert their antimicrobial activity. Because of all the above, further research is needed to include resistant strains to investigate the potential synergistic effect of OLE and standard therapy on the *Candida* species.

According to the available research, no occurrence of resistance to nystatin has been recorded [43], which is in line with the results of this research.

Despite the promising findings and the need to test resistant strains, this study also has several limitations. First, although the disc diffusion method provided valuable insight into the inhibitory effects of extracts, it does not provide detailed information on the rate and extent of microbial killing over time. This uncertainty may prevent a proper understanding of the true antimicrobial potential of OLE. Therefore, future studies in addition to the disk diffusion technique should include methods such as the time–kill assay in order to obtain a more comprehensive insight into the dynamics of antimicrobial activity. Second, our chemical analysis of OLE was performed using high-performance liquid chromatography (HPLC) to confirm the presence of oleuropein and other phenolic compounds. Although HPLC is the standard method for the identification and quantification of these compounds, the lack of liquid chromatography–mass spectrometry (LC–MS) limits the precision in determining the mass and purity of the compounds. This deficiency may lead to ambiguity in the characterization of the active ingredients of OLE, which could affect the validity of our conclusions. In future research, we will use both HPLC and LC–MS techniques to increase the accuracy and reliability of OLE chemical profiling.

## 5. Conclusions

The findings of our study show that OLE has promising in vitro activity against *Candida* species. Our data show that all tested concentrations of OLE (60 µg/µL, 120 µg/µL, 240 µg/µL and 333 µg/µL) have a statistically significant antifungal effect on all *Candida* species, except for the lowest concentration (60 µg/µL). µL) tested on *C. glabrata*. The antifungal effect of OLE was observed to increase with concentration, with 240 µg/µL and 333 µg/µL showing statistically significantly higher antifungal activity compared to the lowest concentration of 60 µg/µL. This indicates a possible potential effect of OLE in treating *Candida*-related oral diseases. Therefore, additional research is needed to investigate the mechanisms of action of OLE and its potential therapeutic effects in vivo as well as optimal doses and methods of administration. The lack of synergistic effects highlights an important area for further research. Given the increasing resistance of *Candida* species to existing antifungal drugs, research into the possibility of OLE working synergistically with other treatments remains crucial. Understanding the interactions between OLE and standard antifungal therapies could lead to new more effective combination treatments that can overcome *Candida* resistance, reduce the incidence of drug side effects and provide better patient outcomes. Investigation of the synergistic potential of OLE with existing antifungal agents should include testing the safety and efficacy of OLE in clinical settings to provide a new approach to treatment, to address the growing problem of antifungal drug resistance and to establish its suitability as a new antifungal therapy for oral cavity diseases caused by *Candida*.

## Figures and Tables

**Table 1:** Concentrations of phenolic compounds in olive leaf extract (OLE) (mg/100 g of dry extract).

	Phenolic Compounds (mg/100 g of Dry Extract)
Oleuropein	17,415.15
Oleacein	1060.1
Verbascoside	668.25
Rutin	227.7
Luteolin-7-*O*-glucoside	2304.9
Apigenin-7-*O*-glucoside	489.1
Luteolin-4-*O*-glucoside	116.55

**Table 2:** The differences in the size of inhibition zones (mm) with regard to the concentrations of OLE.

	Median (Interquartile Range) Inhibition Zone (mm)		Diff. (95% CI)	p *
	DMSO	60 µg/µL		
*C. albicans* (*n* = 36)	6 (6–6)	9.5 (6.13–10.88)	3.5 (1.5–3.5)	<0.001
*C. krusei* (*n* = 16)	6 (6–6)	7.5 (6–10.38)	1.5 (0–4)	0.001
*C. glabrata* (*n* = 5)	6 (6–6.25)	7.5 (6–11)	1.5 (0–5.5)	0.13
*C.tropicalis* (*n* = 3)	6 (6–6)	10.5 (6.5–11)	4.5	0.04
	DMSO	120 µg/µL		
*C. albicans* (*n* = 36)	6 (6–6)	10 (8–11)	4 (2.5–4.5)	<0.001
*C. krusei* (*n* = 16)	6 (6–6)	8.25 (7.5–11.25)	2 (1.5–4.5)	<0.001
*C. glabrata* (*n* = 5)	6 (6–6.25)	9 (7.25–10.25)	3 (1–4.5)	0.007
*C. tropicalis* (*n* = 3)	6 (6–6)	8.5 (8–11)	2.5	0.04
	DMSO	240 µg/µL		
*C. albicans* (*n* = 36)	6 (6–6)	10 (8.5–12)	4 (3–5.5)	<0.001
*C. krusei* (*n* = 16)	6 (6–6)	9.5 (8.5–11.38)	3.5 (2.5–5)	<0.001
*C. glabrata* (*n* = 5)	6 (6–6.25)	9.5 (8–11)	3.5 (2–5)	0.01
*C. tropicalis* (*n* = 3)	6 (6–6)	9.5 (9–10)	3.5	0.04
	DMSO	333 µg/µL		
*C. albicans* (*n* = 36)	6 (6–6)	10.25 (9.5–11.5)	4 (4–4.5)	<0.001
*C. krusei* (*n* = 16)	6 (6–6)	10.25 (9.5–11.5)	4 (3.5–5.5)	<0.001
*C. glabrata* (*n* = 5)	6 (6–6.25)	11 (10–12.25)	5 (4–7)	0.007
*C. tropicalis* (*n* = 3)	6 (6–6)	10 (8.5–10.5)	4.0	0.04

* Mann–Whitney U test.

**Table 3:** Inhibition diameter zones (mm) of different OLE concentrations.

	Median (Interquartile Range) Inhibition Zone (mm)								p *
	60 µg/µL	*p* *	120 µg/µL	*p* *	240 µg/µL	*p* *	333 µg/µL	*p* *	
*C. albicans* (*n* = 36)	9.5 (6.13–10.88)	0.79	10 (8–11)	0.68	10 (8.5–12)	0.82	10.25 (9.5–11.5)	0.79	0.006 ^†^
*C. krusei* (*n* = 16)	7.5 (6–10.38)		8.25 (7.5–11.25)		9.5 (8.5–11.38)		10.25 (9.5–11.5)		0.02 ^†^
*C. glabrata* (*n* = 5)	7.5 (6–11)		9 (7.25–10.25)		9.5 (8–11)		11 (10–12.25)		0.16
*C. tropicalis* (*n* = 3)	10.5 (6.5–11)		8.5 (8–11)		9.5 (9–10)		10 (8.5–10.5)		0.94

* Kruskal–Wallis test (Post hoc Conover) (differences between *Candida* spp.). ^†^ at the level of *p* < 0.05, there are significantly different 60 µg/µL vs. 240 µg/µL and 333 µg/µL.

**Table 4:** The differences in the size of inhibition zones (mm) with regard to the applied therapy.

	Median (Interquartile Range) Inhibition Zone (mm)				Difference (95% CI)	*p* ^§^
	Miconazole	*p* *	Miconazole + OLE 333 µg/µL	*p* *		
*C. albicans* (*n* = 36)	28.5 (26.5–29.5)	0.17	28.5 (23.63–30.75)	0.51	0 (−2 do 1.5)	0.94
*C. krusei* (*n* = 16)	28.5 (27.5–29)		27.75 (26.13 –32.13)		0 (−2 do 3.5)	0.97
*C. glabrata* (*n* = 5)	30 (29–33)		29.5 (25.25–30.75)		−1.5 (−7 do −1.5)	0.29
*C. tropicalis* (*n* = 3)	28.5 (20–29)		24 (20–27.5)		−1.5	0.38

* Kruskal–Wallis test (Post hoc Conover) (differences between *Candida* spp.). ^§^ Mann–Whitney U test.

**Table 5:** The differences in the size of inhibition zones (mm) with regard to the applied therapy.

	Median (Interquartile Range) Inhibition Zone (mm)				Difference (95% CI)	*p* ^§^
	Nystatin	*p* *	Nystatin + 333 µg/µL	*p* *		
*C. albicans* (*n* = 36)	27.5 (26.5–28)	0.01 ^†^	26.25 (25.5–28.25)	0.51	−1 (−1.5 do 0.5)	0.14
*C. krusei* (*n* = 16)	27.5 (27–28)		27.5 (25.5–31.63)		0 (−1.5 do 3)	0.86
*C. glabrata* (*n* = 5)	28 (27–31)		31 (28–35.5)		2.5 (−2.5 do 8)	0.21
*C. tropicalis* (*n* = 3)	25.5 (25.5–26.5)		24.5 (19–29.5)		−1	0.51

* Kruskal–Wallis test (Post hoc Conover) (differences between *Candida* spp.); ^§^ Mann–Whitney U test. ^†^ at the level of *p* < 0.05, there are significantly higher values of *C. glabrata* vs. *C. tropicalis.*

## Data Availability

The data presented in this study are available on request from the corresponding author. The data are not publicly available due to ethical issues.
